# Radiation-Induced Caries: Exploring the Pathway to Manage the Challenge

**DOI:** 10.7759/cureus.76810

**Published:** 2025-01-02

**Authors:** Sara El Harram, Tarik Sqalli

**Affiliations:** 1 Department of Dental Sciences, Faculty of Medicine, Pharmacy and Dentistry, Sidi Mohamed Ben Abdellah University in Fes, Fes, MAR; 2 Laboratory of Epidemiology and Research in Health Sciences, Faculty of Medicine, Pharmacy and Dentistry, Sidi Mohamed Ben Abdellah University in Fes, Fes, MAR

**Keywords:** caries management, etiopathogenesis of radiation caries, head and neck radiotherapy, radiation-induced caries, radiotherapy-induced dental damage

## Abstract

Radiation-induced caries represent a frequent and serious complication of head and neck radiotherapy, significantly affecting patients' quality of life. Their development results from several factors, including the direct effects of radiotherapy on dental tissues, as well as indirect effects related to xerostomia and changes in saliva. Diagnosis is based on clinical and radiographic assessment, which often reveals a specific pattern of demineralization.

The management of these caries requires a comprehensive approach, combining prevention, treatment of lesions, and regular follow-up. It is essential to establish long-term fluoride prophylaxis, and advice on oral hygiene and nutrition, in order to prevent new lesions. The choice of dental restorations must be considered according to the state of salivation and the caries risk, favoring materials adapted to the specificities of radiotherapy.

## Introduction and background

Head and neck cancers are a major health concern whose management is often complicated due to the functional and aesthetic implications of the tumor in this region. Head and neck radiotherapy (HNRT) plays a crucial role in the treatment regimen for these cancers, with a total dose of 50 to 70 Gy delivered in fractions over four to seven weeks [1]. This therapy presents considerable side effects: nearly all patients who have received radiotherapy to the oral cavity will present one or more complications [2]. Even with intensity-modulated radiotherapy (IMRT), which has reduced the effects, these remain inevitable and, when underestimated, could compromise patients' quality of life temporarily or even permanently [3].

Post-radiation carious lesions, also known as radiation-induced carious lesions, are among the most severe complications of cervicofacial radiotherapy. They affect approximately 29% of patients at the end of radiotherapy and up to 37% two years later [4,5]. Their pathogenesis and the mechanisms behind their aggressive progression remain uncertain and continue to be the focus of studies in the literature. Without timely diagnosis and treatment, these lesions can lead to massive crown destruction. Thus, it would be wise to understand the mechanism of occurrence of these lesions in order to better control their semiology and be able to establish correct management.

## Review

Clinical aspect of radiation-induced carious lesions

**Figure 1:**
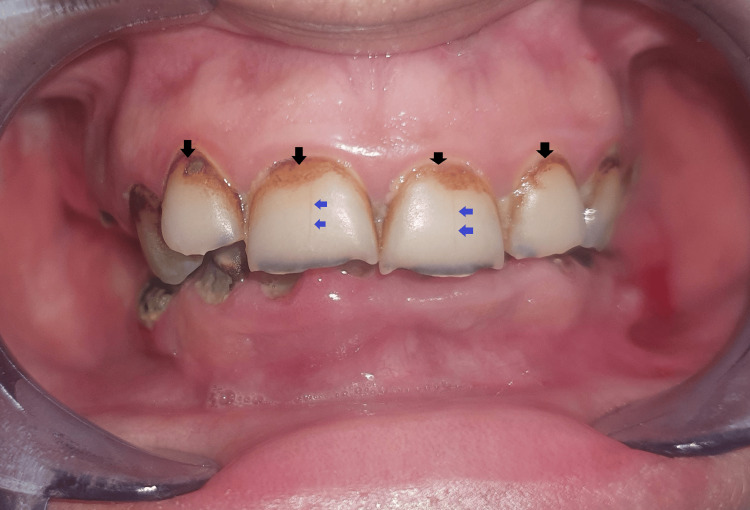
Cervical lesions with hard, smooth surfaces and diffuse brown-to-black discoloration of the enamel (black arrows), an appearance that does not reflect the true extent of the lesion. Enamel fissures (blue arrow) in a patient post-cervico-facial radiotherapy. Image credits: Sara El Harram.

**Figure 2:**
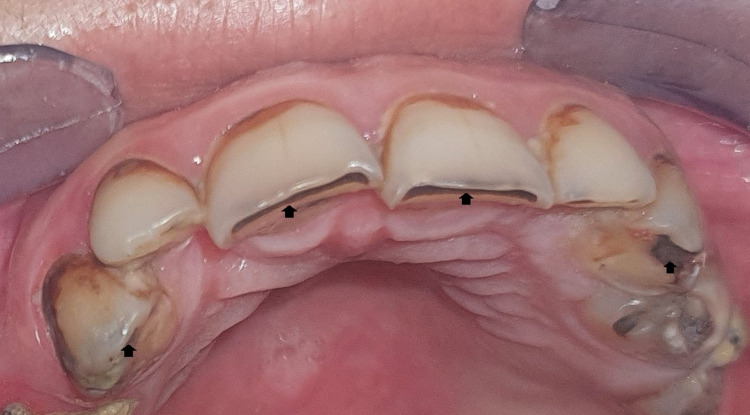
Radiation-induced caries affecting incisal edges and cusp tips (black arrows). Image credits: Sara El Harram.

The radiation-induced carious lesion is characterized by its pattern of onset and progression, as well as its clinical appearance, which are different from those of a conventional carious lesion: Radiation-induced carious lesions begin particularly at the cervical region, the incisal edges, and the cusp tips (Figures 1-2). These lesions initially have a hard and smooth appearance with diffuse brownish-to-black discoloration of the enamel and absence of cavitation (Figure 1) [6,7]. This appearance, which does not accurately reflect the extent of the lesion, can mislead the practitioner. Consequently, he can underestimate the absence of cavitation and omit prompt and appropriate treatment [8].

**Figure 3:**
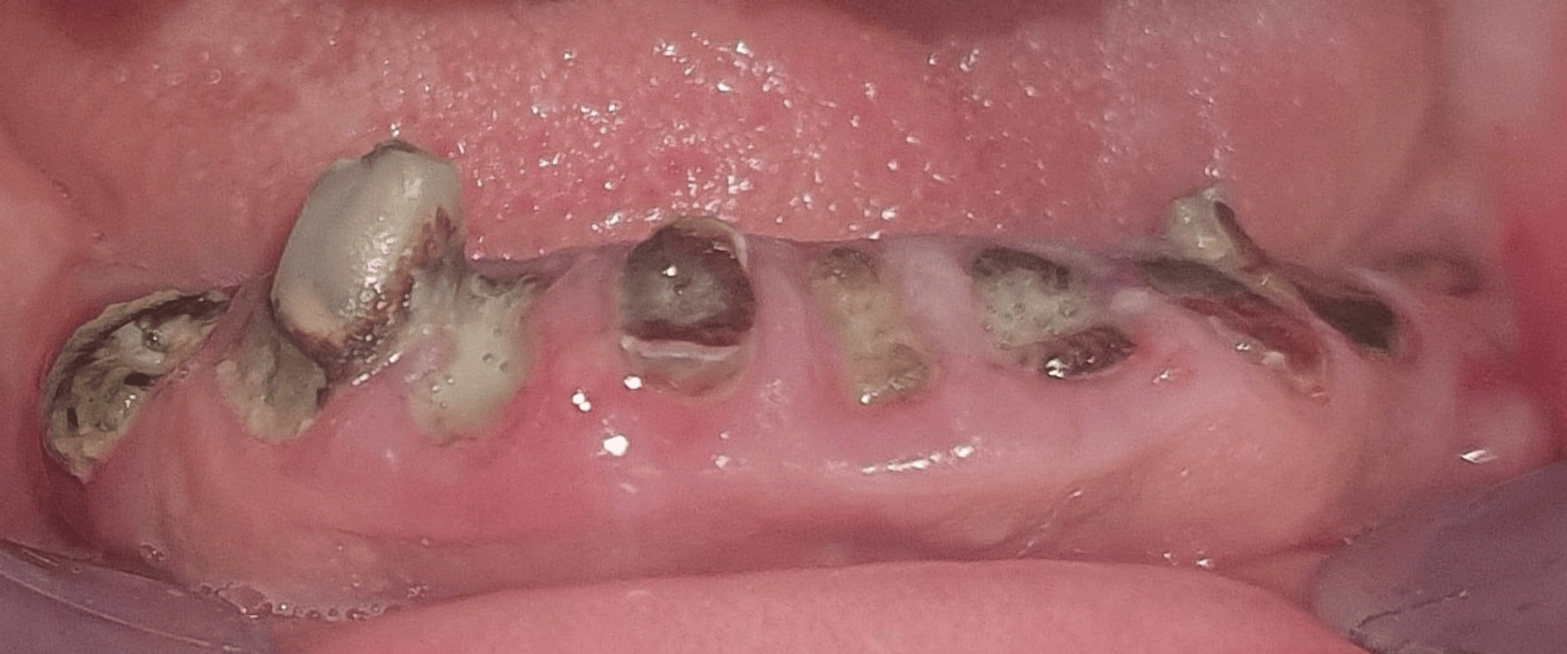
Severe crown destruction resulting from radiation-induced caries in a post-cervico-facial radiotherapy patient. Image credits: Sara El Harram.

The post-radiation carious lesion progresses rapidly through cracked enamel, which has become porous and rough. The enamel eventually fractures, exposing the underlying dentin. The progression continues, leading to massive damage to dental tissues and, thus, crown destruction and amputation (Figure 3) [6-9].

Etiopathogenesis of radiation-induced carious lesions

Currently, it is widely accepted that the development of radiation-induced carious lesions arises from a combination of direct and indirect effects of radiotherapy on the dental organ.

Indirect Effects of Radiotherapy

The presence of salivary glands in the irradiation field can alter their functioning and lead to a quantitative and qualitative change in saliva. By the end of the first week of radiotherapy, the amount of total saliva, both stimulated and at rest, is reduced by 36.67% and 47.9%, respectively [9]. This decrease, causing xerostomia, can progress to asialia. The serous acini, being particularly radiosensitive, undergo a decrease in their activity, which leads to an increase in the saliva's viscosity. Then, it becomes more sticky and adheres to dental and mucosal surfaces. As a result, the ability of saliva to ensure self-cleaning of dental plaque will be affected [10]. The composition of saliva is also modified due to the alteration of membrane ion exchanges, resulting from fibrosis of the walls of the excretory ducts. This alteration causes a decrease in the various salivary constituents (minerals, enzymes, immuno-proteins) and alters the balance of certain electrolytes. These changes reduce the production of antimicrobial components (immunoglobulins A, lysozymes, lactoferrin, peroxidase), as well as a decrease in buffering capacity, leading to a salivary pH drop from seven to five [10]. Furthermore, patients who have undergone cervicofacial radiotherapy frequently suffer from oral mucosa burns and radiomucositis, which complicates tooth brushing and maintaining oral hygiene. This is compounded by the alteration of general health and weight loss, which is associated with the reduction of nutritional intake. Dry mouth and pain in the mucous membranes can also influence food choices, prompting patients to consume cariogenic foods and beverages [11]. These conditions, along with a weakened defense system, lead to an imbalance of the oral flora, favoring the proliferation of cariogenic and periodontopathogenic microorganisms and candida [12]. Moreover, with low salivary pH and buffering capacity of saliva, the dissolution of enamel and dentin minerals becomes easier, while the remineralization process is compromised [13].

Direct Effects of Radiotherapy

Teeth affected by radiation-induced caries (RIC) showed an extensive radiographic pattern of demineralization in the cervical region. These extensive demineralizations can be observed even in teeth with brownish lesions on the enamel surface without cavitation [7], supporting the hypothesis that post-radiation lesions are more destructive than they appear [14]. These teeth show a significant decrease in calcium (Ca) and phosphate (P) levels compared to those with conventional caries and healthy teeth. This finding suggests that the mineral matrix of teeth with RIC has been altered. In addition, a higher amount of carbon has been found in the areas of RIC demineralization [8]. Despite their differences, the conventional and RIC lesions seem to have similar histological patterns [6]. These observations have raised the question of the direct effect of radiotherapy on dental hard tissues. This direct damage has not yet been fully evaluated or elucidated [15,16]. However, the literature has reported some findings that could explain this phenomenon.

The dentinal-enamel junction (DEJ) and inner enamel play a critical role in maintaining the cohesion between enamel and dentin and the absorption of occlusal stresses. A significant reduction in microhardness and elastic modulus has been observed from a cumulative exposure of 30 Gy, particularly in this area, along with the appearance of microcracks in the inner enamel [17-19]. HNRT may alter the micromorphological and biochemical characteristics of the DEJ, likely due to its high organic content [17,20]. Radiation interacts with organic materials and water, inducing free radicals and hydrogen peroxide in dental hard tissues, affecting the proteolysis of collagenous and non-collagenous proteins [21]. It had been suggested that high doses of radiotherapy could activate matrix metalloproteinases (MMPs), particularly MMP-20, which are responsible for the degradation of collagen over several months, leading to protein breakdown and a reduction in the mechanical properties of enamel and dentin. The degeneration of organic matter weakens the interaction between enamel and the DEJ, causing destabilization and exfoliation of the enamel with exposure to the dentin [20].

The decrease in microhardness could also be related to a decarboxylation process of organic materials at the junction between hydroxyapatite crystals and collagen fibers, thereby disrupting their fixation. In addition, the release of carbon dioxide, a by-product of decarboxylation, could contribute to the formation of microcracks in the dental structure [21]. Furthermore, the degeneration of minerals and organic matter induced by radiation could reduce the crystallinity of hydroxyapatite crystals, decreasing their crystallinity and increasing their solubility in acidic pH saliva [18]. All these damages to the microstructure of hard dental tissues create an environment conducive to bacterial adhesion and colonization, which, combined with the indirect effects induced by radiation, increases the risk of post-radiation carious lesions [22,23].

Radiotherapy can also induce a progressive and significant reduction in response to pulp sensitivity tests immediately after treatment or within four to six months following (p < 0.00001). One of the explications of this loss of sensitivity is the presence of vascular lesions in the pulp of irradiated teeth, likely causing ischemia and reducing blood flow, leading to a state of anoxia. Therefore, the myelinated nerve fibers would be inhibited and disrupt sensory perception, even during the progression of carious lesions, which could explain the delayed consultation of patients at an advanced stage of carious lesions or after the appearance of necrosis signs [24].

Preventing and managing carious lesions in patients with orofacial cancers

The treatment of RIC is particularly complex due to the extension and accessibility challenges of the lesions, which can lead to incomplete elimination of carious tissues. Additionally, the cavities are often minimally retentive, complicating restorations. HNRT may also impair the bonding strength of materials and the quality of restorations [25,26]. Management is further complicated by the risk of osteoradionecrosis, which makes extraction decisions more challenging. For these reasons, it is important to implement oral and dental care prior to radiotherapy in order to establish favorable conditions that reduce the risk of radiation-induced carious lesions [26]. The global approach requires, first, periodontal therapy, a key step in reducing bacterial load by removing plaque, thereby creating a healthy gingival environment for restorations. Thereafter, the elimination of all carious lesions and restorations can be performed, ideally with bioactive materials. However, protocols must be adapted to avoid any delays in initiating cancer treatment, which could negatively impact the patient's prognosis. Thus, it may be more relevant to focus on moderate to deep lesions or only symptomatic ones [27,28]. After HNRT, managing RIC lesions has to be a part of a global approach aimed at reducing caries risk (treating existing lesions) and maintaining it at the lowest level possible over the long term (preventing new lesions) [26].

Regardless of the adopted protocol or the timing of the consultation (before or after radiotherapy), it is essential to immediately initiate prophylactic measures aimed at eliminating etiopathogenic factors. The prophylactic phase is based on patient awareness and education in oral health. The purpose is to achieve lasting changes in habits and behaviors. Patient motivation begins with clear information about the side effects of radiotherapy, both immediate (radiomucositis, dysphagia, dysgeusia) and delayed (trismus, aggressive caries, xerostomia). Patients must understand the importance of maintaining rigorous oral hygiene and following a balanced diet to minimize mucositis effects, promote healing, prevent carious lesions and osteoradionecrosis, and ultimately improve their quality of life [29,30].

Long-term prophylaxis is essential for patients treated with HNRT and should ideally be initiated before head and neck treatment. Fluoride helps preserve the mineralization of irradiated enamel and cementum but cannot prevent the alteration of enamel mechanical properties caused by HNRT [31,32]. Despite a decrease in salivary flow, improvements in oral health have been observed, including a reduction in the incidence of RIC and stabilization of cariogenic flora [9,10,33].

Application of 1.1% fluoride gels using vinyl trays for five to 10 minutes daily is recommended, as well as application of 5% fluoride varnish every three months after professional plaque control [9,31,34]. For patients who cannot tolerate trays, a 1.1% (5,000 ppm) fluoride toothpaste is an alternative [29]. Fluoride mouthrinses have also been shown to be effective in preventing caries [35]. However, long-term adherence to these treatments remains a challenge. One study reported that only 12% of patients continued to use fluoride gel with trays after two years [36]. This points to the importance of clinical monitoring and tailoring recommendations to patient needs [28]. The use of a chlorhexidine mouthrinse (0.12% to 0.2%) once or twice daily has been proposed to reduce plaque and *Streptococcus mutans* in patients undergoing HNRT. However, it does not affect *Lactobacillus* or periodontopathogens and may cause side effects such as dental discoloration and temporary taste alterations [28].

Patients should also be given palliative treatment for xerostomia based on patients' needs [37]: saliva substitutes (such as mucin, carboxymethylcellulose, or aloe vera) play a pivotal role in oral lubrication, relieve xerostomia symptoms, and protect enamel while avoiding low pH products that aggravate erosion. For pharmacological stimulation, pilocarpine and cevimeline can improve salivary flow, but their use should be closely monitored, especially in patients with contraindications (respiratory or cardiovascular diseases) [11]. When planning radiotherapy, ideally, the salivary glands should be preserved to minimize the risk of xerostomia. Advanced techniques, such as stem cell therapy, could be considered for severe cases of xerostomia, although further research is needed [10,11].

Nutritional education is essential to prevent excessive consumption of sugary drinks, which is common in xerostomia patients. Sweeteners such as xylitol may be beneficial. A personalized approach should take into account altered food preferences and chewing difficulties, advocating a healthy, less cariogenic diet while maintaining adequate caloric intake [11,38].

Dental restorations in HNRT patients should be adapted to the effects of radiotherapy and specific oral conditions such as xerostomia. Composite resins (CRs) are recommended because of their superior durability compared to glass ionomer cements (GICs) and resin-modified GICs (RMGICs) [28,39,40]. They offer better marginal adaptation and good mechanical resistance but require strict compliance with fluoride use and good oral hygiene to prevent caries recurrence. Conversely, while GICshave recognized cariostatic properties, they are more sensitive to erosion and structural degradation, particularly in the presence of xerostomia [28]. Radiotherapy influences the mechanical behavior of CRs and adhesive systems. Radiotherapy, applied in vitro after bonding procedures, does not significantly modify the bond strength or mechanical behavior of composites. However, if applied before bonding, a reduction in adhesion strength is noted, compromising the longevity of the restoration [26,28]. The choice of materials should, therefore, be guided by the caries risk, salivary status, and fluoride use. For fluoride users, composites and RMGICsoffer better longevity. For non-users, CGIs may be an option to reduce secondary caries, although they require more frequent replacements due to their fragility. Optimal management relies on careful planning, appropriate restoration protocols, and rigorous follow-up (every three months) at a low caries risk, preventing recurrences and promptly detecting potential restoration failures [28,41].

Preventing and managing radiotherapy-induced caries: limitations, recommendations, and future directions

The effects of radiotherapy on dental tissues, particularly regarding the development of radiotherapy-induced caries, remain largely understudied. Several factors limit our understanding and optimal management of these lesions, including limited access to clinical studies specifically dedicated to the direct effects of radiotherapy on dental tissues, as well as the diversity of irradiated patients (variability in administered doses, irradiated areas, and treatment duration). In addition, there is a lack of long-term follow-up to assess the evolution of post-radiotherapy caries lesions and their functional impact.

However, recommendations can be drawn from existing data and clinical practices. The synthesis presented in Table 1 results from an analysis of different studies reviews and highlights the need to develop a standardized protocol for the prevention of radiotherapy-induced caries. Furthermore, regular and multidisciplinary follow-up after radiotherapy, including dentists and oncologists, is strongly recommended to ensure comprehensive patient care [29].

**Table 1:** Dental care sequence for the prevention and treatment of radiation-induced caries in head and neck radiotherapy patients

Dental care step	Details of care
First consultation (before or after head and neck radiotherapy) [29]	Contact interdisciplinary team: cancers’ prognosis/treatment outcomes; type of radiation used/to be utilized; anatomic areas in the radiation field; total dose of radiation; commencement and timeline of head and neck radiotherapy; concurrent therapy (e.g., chemotherapy).
Clinical and radiographical exam [29]	Periodontal charting.
	Identification of soft tissue lesions, xerostomia.
	Documentation of existing restorations.
	Caries charting.
Sensibilization and education of patient [29,30]	Clear information about the side effects of radiotherapy: immediate (radiomucositis, dysphagia, dysgeusia) and delayed (trismus, aggressive caries, xerostomia). Importance of rigorous oral hygiene and a balanced diet to minimize mucositis effects, promote healing, and prevent carious lesions.
	Nutrition education: maintain adequate caloric intake (including foods easy to chew and swallow while limiting sugary and cariogenic foods). Recommend the use of alternative sweeteners (xylitol-containing candy or gum) to avoid excessive consumption of sugary beverages (common in patients with severe xerostomia).
	Xerostomia management: personalized approach is essential to minimize the impact of xerostomia. Palliative treatment: saliva substitutes (e.g., mucin, carboxymethylcellulose, or aloe vera) or, if needed, salivation stimulants (e.g., pilocarpine and cevimeline).
Preventive dental care [33]	Oral hygiene/oral care instructions.
	Long-term fluoride prophylaxis: application of neutral 1.1% sodium fluoride gels with vinyl trays for five to 10 minutes. Application of 5% sodium fluoride varnish every three months after professional plaque control. Fluoridated toothpaste containing 1.1% fluoride (5,000 ppm)+ fluoride-containing mouth rinses for patients who cannot tolerate fluoride trays.
Dental treatment [27,28,39,40]	Hygiene appointment.
	Restorative care: complete elimination of all caries lesion is recommended before radiotherapy but avoid any delays in initiating cancer treatment. Adapt restorative material choice to the effects of radiotherapy and specific oral conditions for fluoride users: composites and resin-modified glass ionomer cements offer better longevity. Composite resins provide better marginal adaptation and superior durability, especially when restorations are performed before radiotherapy, but require strict adherence to fluoride use and good oral hygiene to prevent recurrent caries. For non-users: glass ionomer cements have cariostatic properties but require more frequent replacements due to their fragility, erosion, and structural degradation, particularly in xerostomia patients.
Follow-up and maintenance [29]	Recal exams.
	Treat induced caries lesions. When appropriate, establish ongoing care schedule.
	Ongoing education, monitor oral hygiene, monitor use of fluoride trays.

As for future perspectives, it is essential to conduct longitudinal studies to better understand the impact of radiotherapy on dental microstructure and carious lesions. It is also necessary to conduct more studies on the impact of intraoral radiation stents in the prevention of radiation-induced carious lesions [42]. The development of more effective adhesion protocols or restorative materials for these altered tissues, as well as the exploration of preventive approaches such as remineralizing treatments adapted to irradiated patients, constitute promising paths of research to improve the prevention and management of post-radiotherapy carious lesions.

## Conclusions

RIC represents a major complication of HNRT, with a significant impact on patients' quality of life. Their occurrence results from complex mechanisms combining the indirect and direct effects of radiation on hard dental tissues. The management of these lesions requires a comprehensive approach involving patient education regarding oral and dietary hygiene, fluoride prophylaxis, appropriate restorative treatments, and regular follow-up. By integrating preventive strategies and a multidisciplinary approach, it is possible to reduce radiotherapy-related oral complications and to sustainably improve patients' oral health and general well-being.
